# Notes

**Published:** 1903-01

**Authors:** 


### NOTES.

The sale of the famous pacer "Dan Patch" for the sum of sixty thousand dollars to the International Stock Food Company, is a phenomenal price for even a stallion. He will be continued in the stud and give exhibition miles of speed.

The Western Veterinary College, of Kansas City, Missouri, established by the late Junius H. Wattles, will be maintained by his son, Dr. Junius H. Wattles, Jr., who had been associated with his father in the school since its establishment.

Mr. William C. Whitney provided some time ago a fund to enable the American Museum of Natural History to search the plains of the West for the remains of the primeval horse, and agents of that institution have for the past two years been exploring all the way from Montana to Texas. Many interesting specimens have been found, some of which are now in the museum at New York. The fossil remains of a small herd of the species known as the hipparion were discovered a few months ago, and it is believed that a perfect specimen can be constructed from them.

